# Exposure to childhood maltreatment predicts adult physiological dysregulation, particularly inflammation

**DOI:** 10.1371/journal.pone.0294667

**Published:** 2023-11-30

**Authors:** Jennifer C. Cornman, Jacob Witt, Dana A. Glei, Maxine Weinstein

**Affiliations:** 1 Jennifer C. Cornman Consulting, Columbus, OH, United States of America; 2 Center for Population and Health, Graduate School of Arts and Sciences, Georgetown University, Washington, D.C., United States of America; Universitat der Bundeswehr München: Universitat der Bundeswehr Munchen, GERMANY

## Abstract

Although a growing literature describes the effects of negative childhood experiences on biological outcomes, it is difficult to compare results across studies because of differences in measures of childhood experiences, biological markers, sample characteristics, and included covariates. To ensure comparability across its analyses, this study used a single national survey of adults in the United States—the Midlife in the United States (MIDUS) study—to examine comprehensively the association between adverse childhood experiences, operationalized as childhood maltreatment (CM), and biological markers of risk for poor health and to assess whether these associations differ by type of maltreatment, sex, or race. The sample included 1254, mostly White (78%), adults aged 34–86 years (mean age 57 years), 57% of whom were female. We present incidence rate ratios (IRR) from negative binomial and Poisson regressions to examine the relationships between exposure to CM (emotional, physical, and sexual abuse; emotional and physical neglect; and a CM-index reflecting frequency across all five types of maltreatment) and four biological risk summary scores (overall physiological dysregulation, cardiometabolic risk, inflammation, and hypothalamic pituitary axis/sympathetic nervous system (HPA/SNS) function). We also tested whether the effect of each type of CM varied by sex and by race. The CM-index was associated with higher overall physiological dysregulation and inflammation, but the associations were weaker and not statistically significant for cardiometabolic risk and HPA/SNS function. With the exception of a possible sex difference in the association between sexual abuse and overall physiological dysregulation, there was little evidence that the associations varied systematically by type of CM or by sex or race. We conclude that exposure to CM predicts adult biological risk, particularly inflammation. Inconsistency with previous research suggests that additional research is needed to confirm findings regarding sex and race differences.

## Introduction

A life course perspective on the determinants of adult poor health has indicated that childhood experiences and circumstances can be important factors to consider. The approach posits that the causes of chronic diseases and other health outcomes typically diagnosed in adulthood and old age originate, at least in part, in the experiences and conditions faced earlier in life, including childhood [1]. Previous research has shown that adverse childhood experiences (ACEs), often defined as maltreatment or abuse, are significantly associated with poor physical and mental health outcomes, such as cardiovascular disease [2, 3], mortality [4], respiratory or lung disease [3, 5], and depression [4].

Having established these links, there has been a growing interest in understanding the pathways through which these childhood experiences result in poor health in later life. One possible pathway is the cumulative effect such experiences have on biological processes [1, 6] such as allostatic load–physiological dysregulation–which is thought to reflect physiological wear and tear on the body from the release of hormones during stressful events [7]. These hormones, while critical to allowing the body to process and survive shock in the short term, cause damage and stress that accumulate over time. Understanding the biological consequences of negative childhood experiences not only adds to the understanding of the social determinants of adult health [8] but can also inform both policy and practice for the prevention of these experiences and treatment for those who have had these experiences [6, 9].

Previous research has generally shown that ACEs are associated with higher risk levels for a variety of biological measures, although findings across studies are not always consistent. Two recent reviews of studies of negative childhood experiences and biomarkers of health, one summarizing findings from 40 studies between 2007 and 2017 [9] and the other summarizing findings from 199 studies conducted between 2010 and 2020 [10], report that some studies of childhood adversity and endocrine biomarkers, often cortisol or dehydroepiandrosterone (DHEAS), find a positive relationship while others find a negative relationship. One of the reviews notes that results sometimes depend on whether cortisol levels are based on saliva, urine, or blood [9]. Most of the reviewed studies find that childhood adversity is associated with elevated levels of individual biomarkers that reflect inflammation, such as interleukin 6 and C-reactive protein, but the results are not always significant and can differ depending on the measure of childhood adversity [9, 10]. Similarly, findings are also mixed for biomarkers related to cardiovascular and metabolic function and measures of overall physiological dysregulation [9, 10].

The studies included in these two reviews use various measures of ACEs and biological markers and control for different sets of covariates, making it difficult to identify the source of discrepancies in the results. Two recent studies, however, address some of these issues by examining the association between early life adversity, albeit using two different measures, and biologic risk across multiple physiological systems [11, 12]. Friedman and colleagues [11] examined the association between early life adversity—based on childhood socioeconomic disadvantage (ever on welfare, perceived low income, low educated parents), parental divorce, parental death, and physical abuse—and overall allostatic load as well as seven biological subscales. Their results showed that overall early life adversity (a score ranging from 0–6) is associated with elevated allostatic load and greater inflammation, cardiovascular function, and lipid metabolism, but not with sympathetic or parasympathetic nervous systems, HPA function, or glucose metabolism [11]. Lee and colleagues [12] generated profiles of childhood abuse based on type (emotional abuse, physical abuse, sexual abuse) and frequency of abuse and showed that compared with individuals who did not experience abuse, individuals experiencing more frequent and multifaceted abuse had more imbalanced secretion of stress hormones (i.e., ratios of norepinephrine/cortisol and norepinephrine/epinephrine), higher lipid metabolism scores, and greater overall dysregulation. Cardiovascular function, inflammation and HPA activity were less systematically related to childhood abuse profiles and function of the sympathetic and parasympathetic nervous systems and glucose metabolism were not associated with these profiles of childhood abuse [12].

Even though these two studies incorporated biomarkers of multiple physiological systems, each used a different measure of childhood adversity, highlighting the fact that there is no gold standard for measuring ACEs. ACEs commonly include CM (emotional, physical and sexual abuse; emotional and physical neglect), household dysfunction (e.g., parental substance abuse, parental death, parental divorce), violence (such as witnessing violence or victimization and neighborhood safety), and other experiences such as bullying, economic hardship, and discrimination [13, 14]. Studies vary widely not only in which experiences are included, but also in the measurement of those experiences. For example, some studies have focused on only one adverse experience, such as sexual abuse [15]; others have examined several types of ACEs simultaneously [16]. Further, adverse experiences have been quantified in many ways such as: the presence of any adverse experience; a count of the number of experiences; a dichotomous threshold measure equal to or greater than a certain number of experiences (e.g., four or more); or the severity or frequency of all experiences combined [12–14].

In addition to variations in which biomarkers or ACEs are included, studies also differ with respect to the characteristics of the samples used, often using specialized samples such as college students, female prisoners, nurses, adults with specific disease profiles, or older adults (see review by Deighton and colleagues [9]). As a result, there has been relatively little focus on how the relationships between adverse childhood experiences and biomarkers of health might differ by characteristics such as sex and race, yet the prevalence of childhood adversity and biological risk differ by both sex and race [17–19]. Studies that have analyzed sex or race differences have focused on specific biological systems. For example, one study found that physical and emotional abuse increased metabolic risk for both men and women, but sexual abuse was related to metabolic syndrome only for women [16]. Another examined race differences in the association between an early life adversity (ELA) scale that comprised three types of childhood stressful events and five biomarkers of inflammation and found that experiencing ELA was associated with higher levels of four of the five inflammation markers among African Americans but not among Whites [13].

Our analysis adds to the literature in several ways. First, we use a single national survey of adults in the United States to examine the association between five types of CM (emotional, physical, and sexual abuse, and emotional and physical neglect), the most common measurement of ACEs [14], and biological markers of health that represent function in multiple physiological systems (overall physiological dysregulation, cardiometabolic function, inflammation, and hypothalamic pituitary adrenal axis and sympathetic nervous system function). Using a single data source eliminates issues of comparing results from samples with varying characteristics. Similarly, using consistently measured and constructed indicators and the same control variables across models eliminates sources of discrepancies in findings due to differences in measurement, allowing us to draw more defensible conclusions about the influence of multiple childhood experiences on numerous biological risk factors for poor health later in life. Although Friedman and colleagues [11] performed a similar analysis, they examined a mix of early life adversity indicators, whereas we focus specifically on CM. We also differ from the Lee et al. [12] study because they include only three of the five domains of CM (physical, emotional, sexual abuse) that we include. In addition, we examine several different specifications of CM to test the robustness of results.

Second, we examine whether the associations between CM and biological risk differ by type of CM or by sex and race. While there have been a handful of studies that have tested for sex and race differences in the relationship between CM and biological risk, none that we know of has included the multiple specifications of CM and risk that we are able to include in this study.

## Materials and methods

### Data and sample

The Midlife in the United States study (MIDUS), a longitudinal study first conducted in 1995–96, included non-institutionalized, English-speaking adults aged 25–74 in the continental United States [20]. The main sample, which included an oversample of older adults and men, and a sample of twin pairs was selected using national random digit dialing. The study also included a random sample of the main sample’s siblings and oversamples from five metropolitan areas. These individuals participated in a phone interview and, for those who completed that interview, a mail-in self-administered questionnaire (SAQ) was also given.

We focused on the second wave (MIDUS 2), conducted in 2004–2006, because it included both biomarker and childhood adversity measures. MIDUS 2 included 4,963 respondents (75% of MIDUS 1 survivors) who completed the telephone survey and 4,032 who completed the SAQ ([21, 22]). Of these respondents, 3,191 were eligible (alive in 2004–2005, able to travel to a study clinic, and completed both the telephone interview and the SAQ) to participate in a 2-day visit to a clinical research center for a physical exam; 1,054 participated in the biomarker study [23, 24]. In addition to the reinterview of MIDUS 1 respondents, MIDUS 2 recruited a sample of 838 African Americans living in Milwaukee to enhance studies of Black health. A total of 592 in-person interviews were completed (70.7% response rate) and of these respondents, 398 completed the SAQ and 201 participated in the physical exam [24]. Additional details about eligibility, participation and sample flow are available at http://midus.wisc.edu/findings/Understanding_Data_Collection_in_MIDUS_20220721.pdf.

The physical exam, conducted at the University of California Los Angeles, University of Wisconsin, or Georgetown University, included the collection of a fasting blood specimen before breakfast on the second day of the respondent’s clinic stay; a 12-hour (7 p.m. to 7 a.m.) urine specimen that began on day 1; and measurement of height, weight, waist-hip circumference, and blood pressure ([23]; specimen collection and laboratory protocols are described in https://midus-study.github.io/public-documentation/M2P4/Documentation/M2_Blood_Urine_Saliva_Data_FINAL_20220531.pdf). Each respondent participating in the physical exam also completed an additional self-administered questionnaire that included questions about adverse childhood experiences. We excluded one respondent missing key biomarker measures, resulting in an analytical sample of 1,254. A previous comparison of the MIDUS 2 biomarker sample to the full, national MIDUS 2 sample found that participants in the biomarker component did not significantly differ from the full sample in regard to age, sex, race, marital status, or income, but were significantly more likely to have a college degree and less likely to be current smokers [23].

The MIDUS main study and biomarker study protocols were approved by the Health Sciences Institutional Review board (IRB) of the University of Wisconsin-Madison (#SE-2011-0350 and #H-2008-0060, respectively). The IRBs of the University of California, Los Angeles, and Georgetown University also approved the study protocol. Informed, written consent was obtained from all study participants. MIDUS data are available at https://midus.colectica.org/. Additional study details can be obtained from www.midus.wisc.edu.

### Measures

#### Biological risk

We constructed four summary biomarker scores [25]: an overall physiological dysregulation (PD) score, and scores for cardiometabolic risk, inflammation, and hypothalamic pituitary axis (HPA)/sympathetic nervous system (SNS) function. Employing commonly used practices for measuring biological risk [26], each score was a count of the number of biological markers on which respondents scored in the high-risk range. High-risk was determined from either established clinical cutoff points or the distributions of the biological markers for which established cutoffs were not available [26].

The overall PD score included 18 biological markers. Established clinical cutoff points for high risk were used for systolic (>120mmHg) and diastolic (>80 mmHg) blood pressures (each measure the average of the second and third of three seated readings), total cholesterol (> = 200 mg/dL), high-density lipoprotein (HDL) cholesterol (<40mg/dL), triglycerides (> = 150mg/dL), glycosylated hemoglobin (>7.0>%), body mass index (BMI; <18.5 or > = 30), and high sensitivity C-reactive protein (CRP; >3 mg/L). (See S1 Table for sources, additional details, and descriptive statistics.) For waist-hip ratio, urinary cortisol, norepinephrine, interleukin-6 (IL-6), fibrinogen, soluble intercellular adhesion molecule-1 (sICAM-1), sE-selectin, and soluble IL-6 receptor (sIL-6R), high risk was defined as values greater than or equal to the 75^th^ percentile. High risk for dehydroepiandrosterone-sulfate (DHEA-S) and creatinine clearance (which was derived based on serum creatinine adjusted for sex, age, and body mass using the Cockcroft-Gault equation) was defined as values less than or equal to the 25^th^ percentile. For epinephrine, high risk was defined as values in the top or bottom 12.5th percentile [25, 27]. S2 Table has additional descriptive details about each of these biomarkers.

Cardiometabolic risk counted how many of eight cardiovascular/metabolic markers (systolic and diastolic blood pressure, total cholesterol, HDL cholesterol, triglycerides, glycosylated hemoglobin, BMI, and waist-hip ratio) were high risk. The six biomarkers included in the inflammation score were serum IL-6, CRP, fibrinogen, sICAM-1, sIL-6R, and sE-selectin. Finally, the four markers included in the HPA/SNS risk score were DHEAS and urinary measures of cortisol, epinephrine, and norepinephrine.

#### Childhood maltreatment (CM)

CM was measured using the Childhood Trauma Questionnaire (CTQ; [28]), an inventory of items that measured the frequency of childhood trauma, primarily CM. There were 25 items that comprised five types of CM: emotional abuse, physical abuse, sexual abuse, emotional neglect, and physical neglect [26]. On a scale from 1 (never true) to 5 (very often true), respondents were asked to report how often they experienced certain feelings or events [24]. Sub-indexes using these responses were constructed as outlined in the MIDUS documentation [24], summing the 5 items (recoded to range from 0–4 so that those reporting no maltreatment had a score of 0) that formed each sub-index, capturing the intensity of each type of abuse/neglect (range 0–20). Cronbach’s alphas for the sub-indexes ranged from 0.69 (physical neglect) to 0.93 (sexual abuse). We also constructed a CM-index that reflected the frequency of all types combined (range 0–100; Cronbach’s α = 0.82). In the regression analyses, described below, standardized versions of the indexes were used.

Previous research has suggested that there may be a dose-response relationship between childhood adversity and adult health [9], so we tested an alternative specification of overall CM exposure that counted the number of types of CM experienced. For each of the five types of CM, the respondent was coded as exposed if s/he reported experiencing any of the five items in that sub-index sometimes, often, or very often; those who reported “never” or “rarely” to all items in that sub-index were coded as not exposed. The number of types of maltreatment is the total across the five indicators of any exposure (range 0–5, mean = 1.6). In multivariate analyses, we standardized this CM measure for comparability with other CM measures.

#### Control variables

In addition to self-identified sex (male (43.2%), female (56.8%)) and self-identified race (White (78.0%), Black (18.6%), other (3.2%)), we controlled for potential confounders, including age (using a quadratic specification), childhood socioeconomic status (SES), and a measure that captured the tendency to exaggerate or give desirable answers. We included a quadratic specification for age to capture any non-linear relationships between age and the biomarkers. In cases where the relationship between age and a biomarker score is strictly linear, the quadratic term will be virtually zero (and thus, the results from the model will be essentially the same as if we had omitted the quadratic term). Childhood SES was based on measures of mother’s and father’s educational attainment and an occupational socioeconomic index [29] as well as a perceived financial status question that asked respondents to rate their childhood family’s financial status relative to others (7-point scale ranging from “a lot worse off” to “a lot better off”). These five items were standardized and then averaged to create the childhood SES measure [30]; (Cronbach’s α = 0.76; range -2.4–3.3). The minimization/denial score ranged from 0–3 and counted the number of times a respondent reported that it was “very often true” (vs. never, rarely, sometimes or often true) that “there was nothing I wanted to change about my family”; “I had a perfect childhood”; and “I had the best family in the world” (Cronbach’s α = 0.70; [24]). The average denial score was 0.5 (S3 Table). Previous research has suggested that controlling for the minimization/denial component of the CTQ may reduce the effects of respondents underestimating exposure to childhood trauma [31].

### Analytic strategy

Descriptive characteristics of the analytic sample are presented in the S3 Table. To evaluate the association between overall exposure to CM and the biological summary scores, which are count measures, we estimated a series of negative binomial or Poisson regressions for each of the four biological summary scores. We used Poisson regression for the cardiometabolic and HPA/SNS function scores, but found evidence of over-dispersion (i.e., variation was greater than that of a true Poisson) for the overall PD and inflammation scores. Thus, we used negative binomial regression for those outcomes. For each outcome, we estimated three models. The first model included the CM-index and control variables. The second model included interaction terms between sex and the CM-index to test for sex differences in the association between the CM-index and biological health and a third model included interaction terms between the CM-index and race to test for race differences in the association. Incidence rate ratios (IRR), which are the exponentiated coefficients from the estimated models, are presented. An IRR > 1 indicated a positive association between a predictor and outcome (e.g., an IRR of 2.0 would imply that the incidence rate for the outcome doubles per one-unit increase in the predictor) and an IRR < 1 indicated a negative relationship (e.g., an IRR of 0.5 implies that the incidence rate is halved for every one-unit increase in the predictor).

We tested the sensitivity of these results in two ways. First, in order to understand whether a particular type of CM, such as emotional or sexual abuse, was driving the relationship between CM and biological risk, we estimated a series of models in which each individual CM sub-index was entered in the model rather than the overall CM-index. Each sub-index was also interacted with sex and race. The second set of models assessed whether the individual CM sub-indexes were independently associated with biological risk. In these models, we entered all five sub-indexes simultaneously and subsequently interacted them with sex and race.

All analyses were conducted using Stata 15.1 [32] and most used an alpha < = 0.05 to establish statistically significant findings. Because of the large number of interaction terms estimated in the two sets of analyses just described (9 in the primary analysis, 60 in each of the sensitivity analyses), we applied a Bonferroni adjustment [33] to the interpretation of whether interaction terms were significant. For the primary analysis, the alpha for the interaction terms had to be less than 0.0056 and less than 0.00083 for the sensitivity analyses.

Missing data on analysis variables ranged from 0.6% (physical abuse) to 8.5% (childhood SES). The “mi impute, chained” command in Stata 15.1 [30] was used to perform multiple imputation. For each analysis variable missing data, except race, we used predictive mean matching using the 5 nearest neighbors to impute the missing values. For race, we used a multinomial logistic regression model. We conducted 5 imputations for each variable. All analysis variables were used in the imputation process. IRRs were estimated using the ‘Poisson, irr’ and ‘nbreg, irr’ commands.

## Results

Table 1 examined the relationship between the CM-index and biological markers of health (full models with controls are presented in S4 Table. Also, because there were no significant associations between the CM-index and HPA/SNS function, we also presented those models in S4 Table). The results from Model 1 indicated that higher CM-index scores were significantly associated with higher overall PD (IRR = 1.05 per SD, p<0.001) and greater inflammation (IRR = 1.12 per SD, p<0.001). Results from unadjusted models yielded similar results (see footnote Table 1). This relationship did not differ by sex or by race. None of the interaction terms between sex and the CM-index nor between race and the CM-index was significant at p<0.050 (Models 2 and 3). We also tested the alternative CM measure, ranging from 0–5, that reflected a count of the types of CM that were experienced at least “sometimes” (i.e., for any of the five items that comprised each sub-index). Results showed that the associations between the number of types of CM experienced and each biological risk score (S5 Table) were similar in magnitude to those for the CM-index (Table 1).

**Table 1:** Incidence-rate ratios (standard errors) from negative binomial (NB) or Poisson (P) regression models predicting biomarker health outcomes: Childhood maltreatment (CM)- index (N = 1254)^a^.

	Overall Physiological Dysregulation (NB)			Cardiometabolic Risk (P)				Inflammation (NB)			
VARIABLES	(1)	(2)	(3)		(1)	(2)	(3)		(1)	(2)	(3)	
CM-index^b^^,^^c^	1.05***	1.07**	1.05**		1.03	1.04	1.05*		1.11***	1.17**	1.11**	
	(0.02)	(0.03)	(0.02)		(0.02)	(0.03)	(0.02)		(0.03)	(0.06)	(0.04)	
Female	0.90***	0.90***	0.90***		0.66***	0.66***	0.67***		1.10	1.10	1.10	
	(0.03)	(0.03)	(0.03)		(0.02)	(0.02)	(0.02)		(0.06)	(0.06)	(0.06)	
Female X CM-index^b^		0.98				0.99				0.95		
		(0.03)				(0.04)				(0.05)		
Race (White reference group)												
Black	1.21***	1.21***	1.20***		1.15**	1.15**	1.16**		1.49***	1.48***	1.48****	
	(0.05)	(0.04)	(0.05)		(0.05)	(0.05)	(0.05)		(0.10)	(0.10)	(0.10)	
Other	1.14	1.14	1.19*		1.05	1.05	1.10		1.34*	1.34*	1.40*	
	(0.09)	(0.09)	(0.10)		(0.10)	(0.10)	(0.11)		(0.19)	(0.19)	(0.21)	
Black X CM-index^b^			1.00				0.96				1.01	
			(0.03)				(0.04)				(0.05)	
Other X CM-index^b^			0.87				0.83				0.90	
			(0.07)				(0.09)				(0.13)	

***p<0.001

** p<0.01

* p<0.05

^a^All models controlled for age, age squared, childhood socioeconomic status, and the minimal denial score.

^b^The CM-index was standardized.

^c^IRR for the CM-index for models unadjusted for controls were as follows—Overall physiological dysregulation: 1.04** (0.01); cardiometabolic risk: 1.03 (0.02); inflammation: 1.11***(0.03). See S4 Table for full models including controls.

**Notes:** Models: (1) main effects; (2) main effects + sex interactions; (3) main effects + race interactions

HPA/SNS = Hypothalamic-Pituitary Axis and Sympathetic Nervous system; P = Poisson regression; NB = Negative binomial regression; SES = Socioeconomic status

The results of models that examined each CM-subindex with each measure of biological risk (S6–S9 Tables) showed that all types of abuse except physical neglect were significantly associated with greater overall PD (Models 1, S6 Table). Similarly, all types of abuse except sexual abuse were associated with higher levels of inflammation (S8 Table, Model 1). Only one CM sub-index (emotional abuse) was associated with greater cardiometabolic risk (S7 Table), and none of the CM sub-indexes was associated with the HPA/SNS score (S9 Table, Model 1). These relationships did not differ by sex or race (e.g., none of the interaction terms was significant at the Bonferroni-adjusted alpha p<0.00083).

Results from models that included all five sub-indexes simultaneously (S10 Table) indicated that, when all five CM sub-indexes were entered simultaneously, none of them exhibited a significant independent association (p-values are greater than 0.050) with the biological indicators of health examined here. One interaction term (between sexual abuse and sex in models predicting overall PD) was significant at the Bonferroni-adjusted alpha p<0.00083.

**Fig 1:**
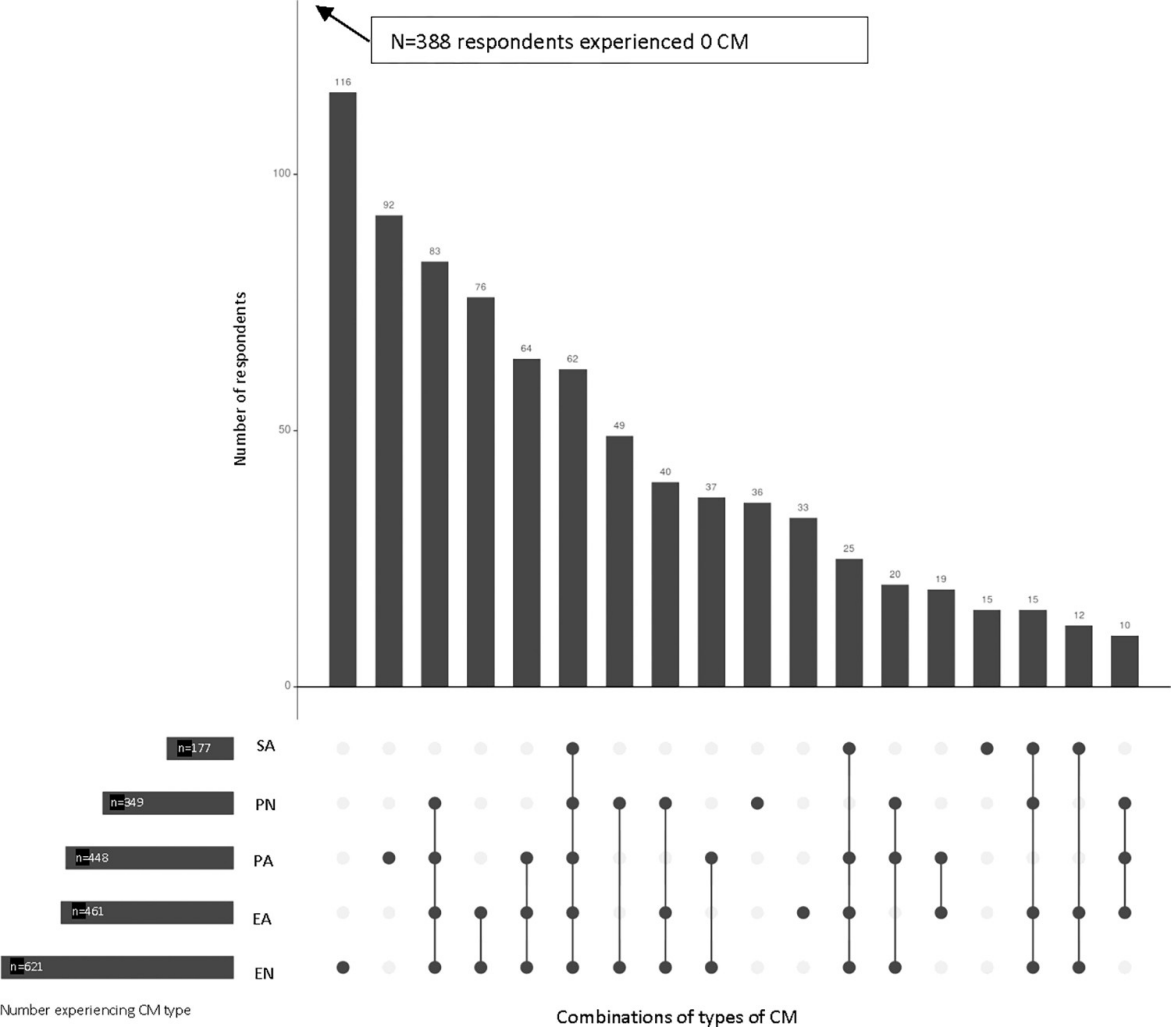
Frequency of combinations of CM types experienced^a^ by 10 or more individuals^b^. Notes: Created in UpSetR [34]. ^a^The respondent is classified as having experienced a given CM type if s/he reports that any of the five feelings/events in that type was experienced at least “sometimes” (as opposed to never or rarely). ^b^An additional 13 patterns each experienced by fewer than 10 individuals (n = 62) are not shown. SA = Sexual abuse, PN = Physical neglect, PA = Physical abuse, EA = Emotional abuse, EN = Emotional neglect.

The result that there were individual but not independent associations between most CM subtypes and the biomarkers may arise from the fact that individuals often experienced more than one type of CM. Fig 1 shows the frequency of CM subtype combinations experienced by respondents. The bars indicate the frequency of each combination. The dots connected with lines below the bars indicate which CM sub-indexes were experienced. The most frequent scenarios were emotional neglect only (n = 116, 13% of those experiencing any CM (n = 866)), followed by physical abuse only (n = 92, 11%). However, CM types were often experienced in combination with at least one other type (n = 574 or 66% of those experiencing any CM). The most frequent combination was emotional abuse, physical abuse, emotional neglect, and physical neglect (n = 83, about 10% of those experiencing any CM), followed by a combination that included emotional abuse and emotional neglect (n = 76, about 9% of those reporting any) and a pattern including emotional abuse, emotional neglect, and physical abuse (n = 64, 7%).

## Discussion

In this study, we have used a single dataset and consistently measured and constructed indicators to assess the association between CM and adult biological risk. We found that the CM was significantly associated with higher overall PD and inflammation, but the relationships with cardiometabolic risk and HPA/SNS function were weak and not significant. In addition, we found little evidence that these associations differed substantially or systematically by type of CM. Most types of abuse were significantly associated with higher PD and inflammation when entered individually, but when all types were included in the same model none was significant and there was little difference in the magnitude of the coefficients. Individual but not independent associations between types of CM and biological risk may have resulted from types of CM often co-occurring with at least one other type of CM. Finally, with the exception of a possible sex difference in the association between sexual abuse and overall PD, we found little evidence that the relationships between CM and biomarkers of health varied systematically by sex or by race.

These results were consistent with, but also differed from, previous research. In comparing our results to two studies that also used MIDUS data and multiple measures of biological risk [11, 12], all three studies found that childhood adversity was associated with higher overall physiological dysregulation. Our CM-index (and a count of the number of types of CM experienced) and the Freidman and colleagues’ [11] measure of the number of early life adverse events experienced were associated with higher inflammation, but the association between CM and inflammation was less clear in Lee et al. [12]. While Friedman and colleagues [11] and Lee and colleagues [12] found evidence for higher cardiometabolic dysfunction for those who experience childhood adversity, our study did not. None of the studies reported an association between childhood adversity and HPA/SNS function. Although there was significant overlap in the biological measures used in these three studies, the measurement of childhood adversity differed in terms of types and frequency of adversity included, suggesting the need for further development of meaningful measures of adverse childhood experiences.

Even though prevalence of adverse childhood experiences and biological risk for poor health differ by sex and race [17–19], we found few studies that examined whether the association between childhood adversity and biological risk differed by these characteristics. Unlike one study [16], which found sex differences in the associations between emotional, physical, and sexual abuse and metabolic syndrome, and another [13], which found race differences in the relationships between biomarkers of inflammation and an early life adversity (ELA) scale comprised of 3 domains of childhood stressful events, we found little evidence that the association between CM and biological risk differs systematically by sex or race. Our results may have differed from the other studies because of differences in the measurement of childhood adversity and biomarkers across the studies. Nonetheless, the fact that our findings were not consistent with previous findings suggests the need for additional research on sex and race disparities in the association between childhood adversity and adult biological risk.

Our study has several strengths. Because we used a single national dataset that included a validated measure of CM sub-types and consistently assayed biomarkers, we could make comparisons across multiple biological systems and multiple types of CM to draw conclusions about the importance of CM for adult biological risk for poor health. Many previous studies have focused on one physiological system or one biomarker and less-detailed measures of CM, making direct comparisons across physiological systems and CM sub-types more difficult. In addition, because sample characteristics, biomarker, CM, and control variables were the same across all of our analyses, we eliminated the sources of discrepancies in findings owing to differences in measurement. Our results, therefore, have provided strong evidence that CM is associated with greater physiological dysregulation that seems to be driven primarily by greater inflammation. Finally, our sample included both men and women and Black and White respondents, allowing us to make direct comparisons between sexes and across racial groups.

This study has several limitations. First, we used retrospective reports of CM, which may be subject to memory problems, recall bias, or affected by current mental health status [14, 35]. Some studies have shown differences in the associations of health outcomes with retrospective vs. prospective reports [35]. Nonetheless, the consistent association between CM (and the number of types of CM experienced) and overall physiological dysregulation and inflammation indicates that recollections and perceptions about childhood are persistent predictors of biological risk. Second, these data did not allow us to assess differences in associations of adversity and biological risk by more refined race-ethnicity groups. While this study did not find differences between Black and White respondents, relationships could differ for Latino or Asian individuals. In addition, results may not be generalizable to the larger US Black population because we included the African American Milwaukee sample. Finally, the measures of childhood adversity that we used primarily reflect CM, which is the most frequent operationalization of childhood adversity [14], but other measures of adversity, such as family dysfunction or conflict, racial discrimination or bullying, as well as patterns of co-occurring adversities may also be important factors to examine [13, 14, 36, 37]. Although we included and viewed childhood SES as a confounding factor (a fundamental cause of other factors, including exposure to CM), other researchers have considered it an additional measure of childhood adversity. A broader definition of childhood adversity, which is beyond the scope of this analysis, might include many of these additional types of adverse experiences.

Our results add to the literature demonstrating that CM is associated with increased biological risk for poor health, particularly inflammation. Although these findings replicate, in part, results from previous research, they are useful because we can more defensibly conclude how CM is related to multiple physiological systems. Although the association between sexual abuse and overall physiological dysregulation appears to differ by sex, this study suggests that the relationships between CM and biological risk do not differ systematically by sex or race, although more research is needed to confirm this finding. In combination with other studies, this research further highlights the need for consensus building around the measurement of childhood adversity, particularly if results are to inform practice and social policy.

## Supporting information

S1 Table Established cutoffs for biomarker measures. (DOCX)

S2 Table Values defining high risk quartiles for biomarkers without established cutoff points. (DOCX)

S3 Table Descriptive statistics for analysis variables (N = 1254). (DOCX)

S4 Table Incidence-rate ratios (standard errors) from negative binomial (NB) or Poisson (P) regression models predicting biomarker health outcomes from models including control variables: Childhood Maltreatment (CM) Index (N = 1254). (DOCX)

S5 Table Incidence-rate ratios (standard errors) from negative binomial (NB) or Poisson (P) regression models predicting biomarker health outcomes from models including control variables: Number of childhood maltreatment (CM) types (N = 1254). (DOCX)

S6 Table Incidence-rate ratios from negative binomial regression models predicting overall physiological dysregulation. (DOCX)

S7 Table Incidence-rate ratios from Poisson regression models predicting cardiometabolic risk. (DOCX)

S8 Table Incidence-rate ratios from negative binomial regression models predicting inflammation. (DOCX)

S9 Table Incidence-rate ratios from Poisson regression models predicting hypothalamic-pituitary axis (HPA) and sympathetic nervous system (SNS) function. (DOCX)

S10 Table Incidence-rate ratios (standard errors) from negative binomial (NB) or Poisson (P) regression models predicting biomarker health outcomes: Childhood maltreatment (CM) sub-indexes (N = 1254). (DOCX)
