# Supplementary material for: Exposure to childhood maltreatment predicts adult physiological dysregulation, particularly inflammation
Source: PLoS One. 2023 Nov 30;18(11):e0294667. doi: 10.1371/journal.pone.0294667 (PMC10688890; doi:10.1371/journal.pone.0294667)
Supplement: S1 Table — (DOCX) [file pone.0294667.s001.docx]

| S1 Table: Established cutoffs for biomarker measures | | | | | |
| --- | --- | --- | --- | --- | --- |
| Biological marker | Cutoff points for high risk | Percent of sample high risk | Interquartile Range (IQR) | Median | Assay method |
| Systolic blood pressure | >120 mmHg^1^ | 70.8 | 118 - 144 | 130 | n/a |
| Diastolic blood pressure | >80 mmHg^2^ | 30.8 | 69 - 82 | 75 | n/a |
| Total cholesterol | >= 200 mg/dL^3^ | 34 | 160 - 213 | 184 | Enzymatic colorimetric |
| High-density lipoprotein (HDL) cholesterol | <40 mg/dL^4^ | 18.4 | 43 - 66 | 53 | Enzymatic colorimetric |
| Triglycerides | >=150 mg/dL^5^ | 28.1 | 77 - 157 | 106 | Enzymatic colorimetric |
| Glycosylated hemoglobin | >7.0%^6^ | 10.4 | 5.6 - 6.2 | 5.9 | Immunoturbidometric |
| Body mass index | <18.5 or >=30^7^ | 41.7 | 25.2 – 33.0 | 28.6 | n/a |
| High sensitivity C-reactive protein | > 3 mg/L^8^ | 2.2 | 0.07 – 0.38 | 0.15 | Immunoelectro- |
|  |  |  |  |  | chemiluminescent |
| [^1^Whelton P, Carey R, Aronow W, et al. 2017 ACC/AHA/AAPA/ABC/ACPM/AGS/APhA/ASH/ASPC/NMA/PCNA Guideline for the Prevention, Detection, Evaluation, and Management of High Blood Pressure in Adults. J Am Coll Cardiol. 2018 May, 71 (19) e127–e248. https://doi.org/10.1016/j.jacc.2017.11.006](file:///C:\Users\OWNER\Downloads\Whelton%20P,%20Carey%20R,%20Aronow%20W,%20et%20al.%202017%20ACC\AHA\AAPA\ABC\ACPM\AGS\APhA\ASH\ASPC\NMA\PCNA%20Guideline%20for%20the%20Prevention,%20Detection,%20Evaluation,%20and%20Management%20of%20High%20Blood%20Pressure%20in%20Adults.%20J%20Am%20Coll%20Cardiol.%202018%20May,%2071%20(19)%20e127–e248.%20https:\doi.org\10.1016\j.jacc.2017.11.006) | | | | | |
| [^2^Whelton P, Carey R, Aronow W, et al. 2017 ACC/AHA/AAPA/ABC/ACPM/AGS/APhA/ASH/ASPC/NMA/PCNA Guideline for the Prevention, Detection, Evaluation, and Management of High Blood Pressure in Adults. J Am Coll Cardiol. 2018 May, 71 (19) e127–e248. https://doi.org/10.1016/j.jacc.2017.11.007](file:///C:\Users\OWNER\Downloads\Whelton%20P,%20Carey%20R,%20Aronow%20W,%20et%20al.%202017%20ACC\AHA\AAPA\ABC\ACPM\AGS\APhA\ASH\ASPC\NMA\PCNA%20Guideline%20for%20the%20Prevention,%20Detection,%20Evaluation,%20and%20Management%20of%20High%20Blood%20Pressure%20in%20Adults.%20J%20Am%20Coll%20Cardiol.%202018%20May,%2071%20(19)%20e127–e248.%20https:\doi.org\10.1016\j.jacc.2017.11.007) | | | | | |
| ^3^Hulley SB. The US National Cholesterol Education Program. Adult treatment guidelines. Drugs. 1988;36 Suppl 3:100-4. doi: 10.2165/00003495-198800363-00021. PMID: 3254822 | | | | | |
| [^4^National Cholesterol Education Program, National Heart, Lung and Blood Institute . (2001). Third report of the national cholesterol education program (NCEP) expert panel on detection, evaluation, and treatment of high blood cholesterol in adults (adult treatment panel III). Bethesda, MD: National Institutes of Health, Report No.: NIH publication no. 01-3670. Retrieved from http://www.nhlbi.nih.gov/guidelines/cholesterol/atp3full.pdf](file:///C:\Users\OWNER\Downloads\National%20Cholesterol%20Education%20Program,%20National%20Heart,%20Lung%20and%20Blood%20Institute%20.%20(2001).%20Third%20report%20of%20the%20national%20cholesterol%20education%20program%20(NCEP)%20expert%20panel%20on%20detection,%20evaluation,%20and%20treatment%20of%20high%20blood%20cholesterol%20in%20adults%20(adult%20treatment%20panel%20III).%20Bethesda,%20MD:%20National%20Institutes%20of%20Health,%20Report%20No.:%20NIH%20publication%20no.%2001-3670.%20Retrieved%20from%20http:\www.nhlbi.nih.gov\guidelines\cholesterol\atp3full.pdf) | | | | | |
| [^5^National Cholesterol Education Program, National Heart, Lung and Blood Institute . (2001). Third report of the national cholesterol education program (NCEP) expert panel on detection, evaluation, and treatment of high blood cholesterol in adults (adult treatment panel III). Bethesda, MD: National Institutes of Health, Report No.: NIH publication no. 01-3670. Retrieved from http://www.nhlbi.nih.gov/guidelines/cholesterol/atp3full.pdf](file:///C:\Users\OWNER\Downloads\National%20Cholesterol%20Education%20Program,%20National%20Heart,%20Lung%20and%20Blood%20Institute%20.%20(2001).%20Third%20report%20of%20the%20national%20cholesterol%20education%20program%20(NCEP)%20expert%20panel%20on%20detection,%20evaluation,%20and%20treatment%20of%20high%20blood%20cholesterol%20in%20adults%20(adult%20treatment%20panel%20III).%20Bethesda,%20MD:%20National%20Institutes%20of%20Health,%20Report%20No.:%20NIH%20publication%20no.%2001-3670.%20Retrieved%20from%20http:\www.nhlbi.nih.gov\guidelines\cholesterol\atp3full.pdf) | | | | | |
| [^6^Gruenewald, T.L., Seeman, T.E., Ryff, C.D., Karlamangla, A.S., & Singer, B.H. (2006). Combinations of biomarkers predictive of later life mortality. PNAS, 103, 14158-14163. DOI: 10.1073/pnas.0606215103](https://doi.org/10.1073/pnas.0606215103) | | | | | |
| [^7^World Health Organization. (2013). BMI classification. Retrieved from http://apps.who.int/bmi/index.jsp?introPage=intro_3.html](file:///C:\Users\OWNER\Downloads\World%20Health%20Organization.%20(2013).%20BMI%20classification.%20Retrieved%20from%20http:\apps.who.int\bmi\index.jsp?introPage=intro_3.html) | | | | | |
| ^8^Pearson, T.A., Mensah, G.A., Alexander, R.W., Anderson, J.L., Cannonill, R.O., Criqui, M, et al. (2003). Markers of inflammation and cardiovascular disease: application to clinical and public health practice: a statement for healthcare professionals from the Centers for Disease Control and Prevention and the American Heart Association. *Circulation, 107,* 499-511. https://doi.org/10.1161/01.CIR.0000052939.59093.45 | | | | | |
