# Supplementary material for: Exposure to childhood maltreatment predicts adult physiological dysregulation, particularly inflammation
Source: PLoS One. 2023 Nov 30;18(11):e0294667. doi: 10.1371/journal.pone.0294667 (PMC10688890; doi:10.1371/journal.pone.0294667)
Supplement: S2 Table — (DOCX) [file pone.0294667.s002.docx]

| S2 Table: Values defining high risk quartiles for biomarkers without established cutoff points | | | | | | |  |
| --- | --- | --- | --- | --- | --- | --- | --- |
| Biomarker | Quartile cutoff | Interquartile Range (IQR) | Median | Assay method |  |  |  |
|  |  |  |  |  |  |  |  |
| Waist-hip ratio | >0.97 | 0.82 – 0.97 | 0.89 | n/a |  |  |  |
| Urinary cortisol | >=19.0 ug/dL | 6.7 – 19.0 | 12.0 | HPLC |  |  |  |
| Urinary Norepinephrine | >=33.3 ng/mL | 18.2 – 33.3 | 25.0 | HPLC-ECD |  |  |  |
| Urinary Epinephrine^a^ | <=0.85 ng/dL or >=3.27 ng/dL | 1.13 – 2.53 | 1.67 | HPLC-ECD |  |  |  |
| Interleukin-6 (IL-6) | >=41.8 pg/mL | 27.0 – 41.8 | 34.1 | ELISA |  |  |  |
| Fibrinogen | >=403.0 mg/dL | 291.0 – 403.0 | 344 | Immunoturbidimetry |  |  |  |
| soluble intercellular adhesion molecule-1 (sICAM-1) | >=338.1 ng/mL | 220.0 – 338.1 | 273.8 | ELISA |  |  |  |
| sE-selectin | >=52.4 ng/mL | 28.2 – 52.4 | 39.1 | ELISA |  |  |  |
| soluble IL-6 receptor (sIL-6R), | >=3.52 pg/mL | 1.37 – 3.52 | 2.17 | ELISA |  |  |  |
| dehydroepiandrosterone-sulfate (DHEA-S) | <=52.0 ug/dL | 52.0 – 145.0 | 87.0 | Immunoelectr-chemiluminescent |  |  |  |
|  |  |  |  |  |  |  |  |
| Abbreviations: ECD=Electrochemical detection ; ELISA=Enzyme Linked Immunosorbent Assay; HPLC=High-pressure liquid chromatography | | | | | | |  |
| ^a^ Goldman, Glei, Lin & Weinstein (2009) and Turra et al. (2005) show that both high and low values of epinephrine are associated with higher mortality, so for epinephrine we use cutoffs based on the top and bottom 12.5% | | | | | | |  |
| Goldman N, Glei DA, Lin Y-H, Weinstein M. Improving mortality prediction using biosocial surveys. Am J Epi. 2009; 169: 769-779. DOI: 10.1093/aje/kwn389 | | | | | | |  |
| Turra CM, Goldman N, Seplaki CL, Glei DA, Lin Y-H, Weinstein M. 2005. Determinants of mortality at older ages: The role of biological markers of chronic disease. Pop Dev Rev. 2005; 31: 677–701. DOI:10.1111/j.1728-4457.2005.00093.x | | | | | | |  |
