# Supplementary material for: Exposure to childhood maltreatment predicts adult physiological dysregulation, particularly inflammation
Source: PLoS One. 2023 Nov 30;18(11):e0294667. doi: 10.1371/journal.pone.0294667 (PMC10688890; doi:10.1371/journal.pone.0294667)
Supplement: S3 Table — (DOCX) [file pone.0294667.s003.docx]

| S3 Table: Descriptive statistics for analysis variables (N=1254) | | | |
| --- | --- | --- | --- |
|  | Percent/ Mean (std dev) | Median | IQR |
| Biomarker scores (theoretical range) |  |  |  |
| Physiological Dysregulation (0-18) | 4.9 (2.5) | 5 | 3-6 |
| Missing (%) | 0.08 |  |  |
| Cardiometabolic risk (0-9) | 2.6 (1.7) | 2 | 1-4 |
| Missing (%) | 0.08 |  |  |
| Inflammation (0-5) | 1.3 (1.2) | 1 | 0-2 |
| HPA/SNS function (0-4) | 1.0 (0.9) | 1 | 0-2 |
| Childhood maltreatment (CM; theoretical range) | |  |  |
| Emotional Abuse (0-20) | 3.0 (4.2) | 1 | 0-4 |
| Missing (%) | 1.1 |  |  |
| Physical Abuse (0-20) | 2.0 (3.0) | 1 | 0-2 |
| Missing (%) | 0.6 |  |  |
| Sexual Abuse (0-20) | 1.6 (4.0) | 0 | 0 |
| Missing (%) | 1.4 |  |  |
| Emotional Neglect (0-20) | 4.8 (4.6) | 4 | 1-8 |
| Missing (%) | 1.4 |  |  |
| Physical Neglect (0-20) | 1.9 (2.7) | 1 | 0-3 |
| Missing (%) | 1.4 |  |  |
| Total severity of CM (0-100) | 13.0 (14.1) | 8 | 3-18 |
| Missing (%) | 2.9 |  |  |
| Demographic characteristics |  |  |  |
| Age (34 - 86) | 56.9 (11.8) | 56 | 48-65 |
| Female (%) | 56.8 |  |  |
|  |  |  |  |
| White (%) | 78.0 |  |  |
| Black (%) | 18.6 |  |  |
| Other (%) | 3.2 |  |  |
| Missing (%) | 0.2 |  |  |
| Childhood socioeconomic status | 0 (1.0) | -0.1 | -0.7 - 0.6 |
| Missing (%) | 8.5 |  |  |
| Minimum denial score (0-3) | 0.5 (0.9) | 0 | 0 - 1 |
|  |  |  |  |
| Notes: HPA = hypothalamic pituitary adrenal axis; SNS=sympathetic nervous system | | | |
| IQR = inter-quartile range |  |  |  |
