# Supplementary material for: Exposure to childhood maltreatment predicts adult physiological dysregulation, particularly inflammation
Source: PLoS One. 2023 Nov 30;18(11):e0294667. doi: 10.1371/journal.pone.0294667 (PMC10688890; doi:10.1371/journal.pone.0294667)
Supplement: Supplementary file 4 [file pone.0294667.s004.docx]

Table S4: Incidence-rate ratios (standard errors) from negative binomial (NB) or Poisson (P) regression models predicting biomarker health outcomes from models including control variables: Childhood Maltreatment (CM) Index (N=1254)

	Overall Physiological Dysregulation (NB)			Cardiometabolic Risk (P)			Inflammation (NB)			HPA/SNS Function (P)		
VARIABLES	(1)	(2)	(3)	(1)	(2)	(3)	(1)	(2)	(3)	(1)	(2)	(3)
CM index[a]	1.05***	1.07**	1.05**	1.03	1.04	1.05*	1.11***	1.17**	1.11**	1.01	1.00	0.99
	(0.02)	(0.03)	(0.02)	(0.02)	(0.03)	(0.02)	(0.03)	(0.06)	(0.04)	(0.03)	(0.06)	(0.04)
Female	0.90***	0.90***	0.90***	0.66***	0.66***	0.67***	1.10	1.10	1.10	1.54***	1.54***	1.54***
	(0.03)	(0.03)	(0.03)	(0.02)	(0.02)	(0.02)	(0.06)	(0.06)	(0.06)	(0.09)	(0.09)	(0.09)
Female X CM index[a]		0.98			0.99			0.95			1.00	
		(0.03)			(0.04)			(0.05)			(0.07)	
Race (White omitted)												
Black	1.21***	1.21***	1.20***	1.15**	1.15**	1.16**	1.49***	1.48***	1.48***	0.98	0.98	0.96
	(0.04)	(0.04)	(0.05)	(0.05)	(0.05)	(0.05)	(0.10)	(0.10)	(0.10)	(0.08)	(0.08)	(0.08)
Other	1.14	1.14	1.19*	1.05	1.05	1.10	1.34*	1.34*	1.40*	1.14	1.14	1.16
	(0.09)	(0.09)	(0.10)	(0.10)	(0.10)	(0.11)	(0.19)	(0.19)	(0.21)	(0.18)	(0.18)	(0.20)
Black X CM index[a]			1.00			0.96			1.01			1.08
			(0.03)			(0.04)			(0.05)			(0.07)
Other X CM index[a]			0.87			0.83			0.90			0.97
			(0.07)			(0.09)			(0.13)			(0.16)
Controls												
Age[c]	1.01	1.01	1.01	1.03*	1.03*	1.03*	0.97	0.97	0.97	1.03	1.03	1.03
	(0.01)	(0.01)	(0.01)	(0.01)	(0.01)	(0.01)	(0.02)	(0.02)	(0.02)	(0.02)	(0.02)	(0.02)
Age squared[c]	1.00	1.00	1.00	1.00*	1.00*	1.00*	1.00*	1.00*	1.00*	1.00	1.00	1.00
	(0.00)	(0.00)	(0.00)	(0.00)	(0.00)	(0.00)	(0.00)	(0.00)	(0.00)	(0.00)	(0.00)	(0.00)
Childhood SES	0.95**	0.95**	0.95**	0.93**	0.93**	0.93**	0.97	0.97	0.97	0.96	0.96	0.96
	(0.02)	(0.02)	(0.02)	(0.02)	(0.02)	(0.02)	(0.03)	(0.03)	(0.03)	(0.03)	(0.03)	(0.03)
Minimal denial score	1.02	1.02	1.02	1.00	1.00	1.00	1.05	1.05	1.05	1.02	1.02	1.02
	(0.02)	(0.02)	(0.02)	(0.02)	(0.02)	(0.02)	(0.03)	(0.03)	(0.03)	(0.03)	(0.03)	(0.03)
Constant	2.90***	2.94***	2.95***	1.19	1.19	1.23	1.97	2.02	1.99	0.18**	0.18**	0.18**
	(0.90)	(0.91)	(0.91)	(0.47)	(0.47)	(0.49)	(1.12)	(1.15)	(1.13)	(0.12)	(0.11)	(0.11)

*** p<0.001, ** p<0.01, * p<0.05

[a] The CM index has been standardized.

[b] IRR for the CM index for models unadjusted for controls were as follows - Overall physiological dysregulation: 1.04** (0.01); cardiometabolic risk: 1.03 (0.02); inflammation: 1.11***(0.03); and HPA/SNS function: 0.99 (0.03)

[c] For overall physiological dysregulation, cardiometabolic risk, and HPA/SNS function, the coefficients (not shown) imply that dysregulation increases with age up to a point and then declines at older ages (i.e., the coefficient for the linear age term is positive (IRR>1.0) but the coefficient for the quadratic age term is negative (IRR<1.0)). In the case of inflammation, the coefficient for the linear age term is negative (IRR<1.0) but the coefficient for the quadratic age term is positive (IRR>1.0), implying that inflammation decreases with age to a point but increases after that.

Notes: HPA/SNS=Hypothalamic-Pituitary Axis and Sympathetic Nervous system; SES=Socioeconomic status

Models: (1) main effects; (2) main effects + sex interactions; (3) main effects + race interactions
