# Supplementary material for: Exposure to childhood maltreatment predicts adult physiological dysregulation, particularly inflammation
Source: PLoS One. 2023 Nov 30;18(11):e0294667. doi: 10.1371/journal.pone.0294667 (PMC10688890; doi:10.1371/journal.pone.0294667)
Supplement: Supplementary file 5 [file pone.0294667.s005.docx]

S5 Table: Incidence-rate ratios (standard errors) from negative binomial (NB) or Poisson (P) regression models predicting biomarker health outcomes from models including control variables: Number of childhood maltreatment (CM) types (N=1254)

	Overall Physiological			Cardiometabolic Risk			Inflammation			HPA/SNS Function		
VARIABLES	(1)	(2)	(3)	(1)	(2)	(3)	(1)	(2)	(3)	(1)	(2)	(3)
Number of CM types[a,b]	1.05**	1.05*	1.05**	1.03	1.02	1.05	1.11***	1.15**	1.10**	1.02	1.00	1.01
	(0.02)	(0.03)	(0.02)	(0.02)	(0.03)	(0.02)	(0.03)	(0.06)	(0.04)	(0.03)	(0.06)	(0.04)
Female	0.90***	0.90***	0.90***	0.67***	0.67***	0.67***	1.11	1.11	1.11	1.54***	1.54***	1.54***
	(0.03)	(0.03)	(0.03)	(0.02)	(0.02)	(0.02)	(0.06)	(0.06)	(0.06)	(0.09)	(0.09)	(0.09)
Female X Number CM types[a],		0.99			1.01			0.95			1.03	
		(0.03)			(0.04)			(0.05)			(0.07)	
Race (White omitted)												
Black	1.20***	1.20***	1.20***	1.15**	1.15**	1.16**	1.47***	1.47***	1.47***	0.97	0.97	0.96
	(0.05)	(0.05)	(0.05)	(0.05)	(0.05)	(0.06)	(0.10)	(0.10)	(0.10)	(0.08)	(0.08)	(0.08)
Other	1.13	1.14	1.17	1.05	1.05	1.10	1.33*	1.34*	1.33	1.12	1.11	1.13
	(0.09)	(0.09)	(0.10)	(0.10)	(0.10)	(0.11)	(0.19)	(0.19)	(0.22)	(0.18)	(0.18)	(0.20)
Black X Number CM types[a]			0.99			0.94			1.02			1.06
			(0.03)			(0.04)			(0.06)			(0.08)
Other X Number CM types[a]			0.90			0.82			1.01			0.98
			(0.08)			(0.09)			(0.16)			(0.17)
Age[c]	1.01	1.01	1.01	1.03*	1.03*	1.03*	0.97	0.97	0.97	1.03	1.03	1.03
	(0.01)	(0.01)	(0.01)	(0.01)	(0.01)	(0.01)	(0.02)	(0.02)	(0.02)	(0.02)	(0.02)	(0.02)
Age squared[c]	1.00	1.00	1.00	1.00*	1.00*	1.00*	1.00*	1.00*	1.00*	1.00	1.00	1.00
	(0.00)	(0.00)	(0.00)	(0.00)	(0.00)	(0.00)	(0.00)	(0.00)	(0.00)	(0.00)	(0.00)	(0.00)
Childhood SES	0.95**	0.95**	0.95**	0.94**	0.94**	0.94***	0.97	0.97	0.97	0.96	0.96	0.96
	(0.02)	(0.02)	(0.02)	(0.02)	(0.02)	(0.02)	(0.03)	(0.03)	(0.03)	(0.03)	(0.03)	(0.03)
Minimum denial	1.02	1.02	1.02	1.00	1.00	1.00	1.05	1.05	1.05	1.03	1.03	1.03
	(0.02)	(0.02)	(0.02)	(0.02)	(0.02)	(0.02)	(0.03)	(0.03)	(0.03)	(0.03)	(0.03)	(0.03)
Constant	2.85***	2.87***	2.86***	1.18	1.17	1.20	1.93	1.97	1.91	0.18**	0.18**	0.18**
	(0.89)	(0.89)	(0.89)	(0.47)	(0.46)	(0.47)	(1.10)	(1.12)	(1.09)	(0.11)	(0.11)	(0.11)
Observations	1,254	1,254	1,254	1,254	1,254	1,254	1,254	1,254	1,254	1,254	1,254	1,254

*** p<0.001, ** p<0.01, * p<0.05

[a]For each of the five types of childhood maltreatment, any exposure to the type reflects whether any of the five items in the type index was experienced at least "sometimes". Number of types of maltreatment is the total across these five indicators of any exposure. This CM measure has been standardized for comparability with other CM measures.

[b] IRR for the CM index for models unadjusted for controls were as follows - Overall physiological dysregulation: 1.04** (0.02); cardiometabolic risk: 1.03 (0.02); inflammation: 1.11***(0.03); and HPA/SNS function: 0.99 (0.03)

[c] For overall physiological dysregulation, cardiometabolic risk, and HPA/SNS function, the coefficients (not shown) imply that dysregulation increases with age up to a point and then declines at older ages (i.e., the coefficient for the linear age term is positive (IRR>1.0) but the coefficient for the quadratic age term is negative (IRR<1.0)). In the case of inflammation, the coefficient for the linear age term is negative (IRR<1.0) but the coefficient for the quadratic age term is positive (IRR>1.0), implying that inflammation decreases with age to a point but increases after that.

Notes: HPA/SNS=Hypothalamic-Pituitary Axis and Sympathetic Nervous system; SES=Socioeconomic status
Models: (1) main effects; (2) main effects + sex interactions; (3) main effects + race interactions
