# Supplementary material for: Exposure to childhood maltreatment predicts adult physiological dysregulation, particularly inflammation
Source: PLoS One. 2023 Nov 30;18(11):e0294667. doi: 10.1371/journal.pone.0294667 (PMC10688890; doi:10.1371/journal.pone.0294667)
Supplement: Supplementary file 7 [file pone.0294667.s007.docx]

S7 Table : Incidence-rate ratios from Poisson regression models predicting cardiometabolic risk

| | Type-of-Childhood-Maltreatment-Specific Models[b] | | | | | | | | | | | | | | |
|---|---|---|---|---|---|---|---|---|---|---|---|---|---|---|---|
| | CM = EMOTIONAL ABUSE | | | CM = PHYSICAL ABUSE | | | CM = SEXUAL ABUSE | | | CM = EMOTIONAL NEGLECT | | | CM = PHYSICAL NEGLECT | | |
| | (1) | (2) | (3) | (1) | (2) | (3) | (1) | (2) | (3) | (1) | (2) | (3) | (1) | (2) | (3) |
| CM sub-index[a] | 1.04* | 1.07* | 1.05* | 1.03 | 1.05 | 1.06** | 1.03 | 0.98 | 1.03 | 1.02 | 1.03 | 1.04 | 1.01 | 0.99 | 1.02 |
| | (0.02) | (0.03) | (0.02) | (0.02) | (0.03) | (0.02) | (0.02) | (0.04) | (0.02) | (0.02) | (0.03) | (0.02) | (0.02) | (0.03) | (0.02) |
| Female | 0.66*** | 0.66*** | 0.66*** | 0.67*** | 0.67*** | 0.67*** | 0.66*** | 0.67*** | 0.66*** | 0.67*** | 0.67*** | 0.67*** | 0.67*** | 0.67*** | 0.67*** |
| | (0.02) | (0.02) | (0.02) | (0.02) | (0.02) | (0.02) | (0.02) | (0.02) | (0.02) | (0.02) | (0.02) | (0.02) | (0.02) | (0.02) | (0.02) |
| Female X CM sub-index[a] | | 0.96 | | | 0.96 | | | 1.07 | | | 0.99 | | | 1.04 | |
| | | (0.03) | | | (0.03) | | | (0.05) | | | (0.03) | | | (0.04) | |
| Race (White omitted) | 1.16** | 1.16** | 1.16** | 1.15** | 1.15** | 1.17*** | 1.16** | 1.15** | 1.15** | 1.16** | 1.16** | 1.16** | 1.15** | 1.16** | 1.16** |
| Black | (0.05) | (0.05) | (0.05) | (0.05) | (0.05) | (0.06) | (0.05) | (0.05) | (0.05) | (0.05) | (0.05) | (0.05) | (0.05) | (0.05) | (0.06) |
| | 1.05 | 1.05 | 1.07 | 1.05 | 1.05 | 1.07 | 1.05 | 1.05 | 1.06 | 1.05 | 1.05 | 1.09 | 1.05 | 1.05 | 1.10 |
| Other | (0.10) | (0.10) | (0.11) | (0.10) | (0.10) | (0.11) | (0.10) | (0.10) | (0.10) | (0.10) | (0.10) | (0.11) | (0.10) | (0.10) | (0.11) |
| Black X CM sub-index[a] | | | 0.97 | | | 0.92* | | | 1.01 | | | 0.98 | | | 0.97 |
| | | | (0.04) | | | (0.04) | | | (0.04) | | | (0.04) | | | (0.04) |
| Other X CM sub-index[a] | | | 0.91 | | | 0.90 | | | 0.86 | | | 0.86 | | | 0.85 |
| | | | (0.09) | | | (0.11) | | | (0.11) | | | (0.08) | | | (0.08) |
| Age[c] | 1.03* | 1.03* | 1.03* | 1.03* | 1.03* | 1.03* | 1.03* | 1.03* | 1.03* | 1.03* | 1.03* | 1.03* | 1.03* | 1.03* | 1.03* |
| | (0.01) | (0.01) | (0.01) | (0.01) | (0.01) | (0.01) | (0.01) | (0.01) | (0.01) | (0.01) | (0.01) | (0.01) | (0.01) | (0.01) | (0.01) |
| Age squared[c] | 1.00* | 1.00* | 1.00* | 1.00* | 1.00* | 1.00* | 1.00* | 1.00* | 1.00* | 1.00* | 1.00* | 1.00* | 1.00* | 1.00* | 1.00* |
| | (0.00) | (0.00) | (0.00) | (0.00) | (0.00) | (0.00) | (0.00) | (0.00) | (0.00) | (0.00) | (0.00) | (0.00) | (0.00) | (0.00) | (0.00) |
| Childhood SES | 0.94** | 0.94*** | 0.94*** | 0.94** | 0.94*** | 0.94** | 0.94*** | 0.94*** | 0.94*** | 0.94*** | 0.94*** | 0.93*** | 0.93*** | 0.93*** | 0.93*** |
| | (0.02) | (0.02) | (0.02) | (0.02) | (0.02) | (0.02) | (0.02) | (0.02) | (0.02) | (0.02) | (0.02) | (0.02) | (0.02) | (0.02) | (0.02) |
| Minimal denial | 1.00 | 1.00 | 1.00 | 0.99 | 0.99 | 0.99 | 0.99 | 0.99 | 0.99 | 1.00 | 1.00 | 1.00 | 0.99 | 0.99 | 0.99 |
| | (0.02) | (0.02) | (0.02) | (0.02) | (0.02) | (0.02) | (0.02) | (0.02) | (0.02) | (0.02) | (0.02) | (0.02) | (0.02) | (0.02) | (0.02) |
| Constant | 1.17 | 1.19 | 1.19 | 1.18 | 1.19 | 1.19 | 1.17 | 1.15 | 1.16 | 1.19 | 1.20 | 1.23 | 1.19 | 1.18 | 1.21 |
| | (0.46) | (0.47) | (0.47) | (0.47) | (0.47) | (0.47) | (0.46) | (0.46) | (0.46) | (0.47) | (0.47) | (0.49) | (0.47) | (0.47) | (0.48) |
| Observations | 1,254 | 1,254 | 1,254 | 1,254 | 1,254 | 1,254 | 1,254 | 1,254 | 1,254 | 1,254 | 1,254 | 1,254 | 1,254 | 1,254 | 1,254 |

*p<0.05, **p<0.01, ***p<0.001

CM=childhood maltreatment

[a] CM sub-indexes have been standardized

[b] Each set of columns comprises models that include only the CM sub-index given in the column heading.

[c] For overall physiological dysregulation, cardiometabolic risk, and HPA/SNS function, the coefficients (not shown) imply that dysregulation increases with age up to a point and then declines at older ages (i.e., the coefficient for the linear age term is positive (IRR>1.0) but the coefficient for the quadratic age term is negative (IRR<1.0)). In the case of inflammation, the coefficient for the linear age term is negative (IRR<1.0) but the coefficient for the quadratic age term is positive (IRR>1.0), implying that inflammation decreases with age to a point but increases after that.

Notes: SES=Socioeconomic status; Models: (1) main effects; (2) main effects + sex interactions; (3) main effects + race interactions
