# Supplementary material for: Exposure to childhood maltreatment predicts adult physiological dysregulation, particularly inflammation
Source: PLoS One. 2023 Nov 30;18(11):e0294667. doi: 10.1371/journal.pone.0294667 (PMC10688890; doi:10.1371/journal.pone.0294667)
Supplement: Supplementary file 9 [file pone.0294667.s009.docx]

S9 Table: Incidence-rate ratios from Poisson regression models predicting hypothalamic-pituitary axis (HPA) and sympathetic nervous system (SNS) function

	Type-of-Childhood-Maltreatment-Specific Models[b]														
	**CM = EMOTIONAL ABUSE**			**CM = PHYSICAL ABUSE**			**CM = SEXUAL ABUSE**			**CM = EMOTIONAL NEGLECT**			**CM = PHYSICAL NEGLECT**		
	(1)	(2)	(3)	(1)	(2)	(3)	(1)	(2)	(3)	(1)	(2)	(3)	(1)	(2)	(3)
CM sub-index[a]	0.99	1.00	0.96	0.98	0.99	0.96	1.03	0.94	1.01	1.03	1.04	1.02	0.99	0.98	0.99
	(0.03)	(0.06)	(0.04)	(0.03)	(0.06)	(0.04)	(0.03)	(0.09)	(0.03)	(0.03)	(0.06)	(0.04)	(0.03)	(0.06)	(0.04)
Female	1.55***	1.54***	1.55***	1.54***	1.54***	1.54***	1.53***	1.56***	1.53***	1.54***	1.54***	1.54***	1.54***	1.54***	1.54***
	(0.09)	(0.09)	(0.09)	(0.09)	(0.09)	(0.09)	(0.09)	(0.10)	(0.09)	(0.09)	(0.09)	(0.09)	(0.09)	(0.09)	(0.09)
Female X CM sub-index[a]		0.99			0.98			1.11			0.98			1.01	
		(0.07)			(0.06)			(0.11)			(0.06)			(0.06)	
Race (White omitted)															
Black	0.98	0.98	0.97	0.98	0.98	0.97	0.98	0.97	0.96	0.98	0.98	0.97	0.98	0.98	0.98
	(0.08)	(0.08)	(0.08)	(0.08)	(0.08)	(0.08)	(0.08)	(0.08)	(0.08)	(0.08)	(0.08)	(0.08)	(0.08)	(0.08)	(0.08)
Other	1.12	1.12	1.12	1.13	1.13	1.12	1.12	1.12	1.16	1.12	1.12	1.13	1.13	1.13	1.11
	(0.18)	(0.18)	(0.18)	(0.18)	(0.18)	(0.18)	(0.18)	(0.18)	(0.18)	(0.18)	(0.18)	(0.19)	(0.18)	(0.18)	(0.19)
Black X CM sub-index[a]			1.12			1.08			1.08			1.02			0.99
			(0.07)			(0.07)			(0.07)			(0.07)			(0.07)
Other X CM sub-index[a]			1.04			1.04			0.77			0.99			1.03
			(0.15)			(0.20)			(0.16)			(0.14)			(0.13)
Age[c]	1.03	1.03	1.03	1.03	1.03	1.03	1.03	1.03	1.03	1.03	1.03	1.03	1.03	1.03	1.03
	(0.02)	(0.02)	(0.02)	(0.02)	(0.02)	(0.02)	(0.02)	(0.02)	(0.02)	(0.02)	(0.02)	(0.02)	(0.02)	(0.02)	(0.02)
Age squared[c]	1.00	1.00	1.00	1.00	1.00	1.00	1.00	1.00	1.00	1.00	1.00	1.00	1.00	1.00	1.00
	(0.00)	(0.00)	(0.00)	(0.00)	(0.00)	(0.00)	(0.00)	(0.00)	(0.00)	(0.00)	(0.00)	(0.00)	(0.00)	(0.00)	(0.00)
Childhood SES	0.96	0.96	0.96	0.96	0.95	0.95	0.96	0.96	0.97	0.96	0.96	0.96	0.96	0.96	0.96
	(0.03)	(0.03)	(0.03)	(0.03)	(0.03)	(0.03)	(0.03)	(0.03)	(0.03)	(0.03)	(0.03)	(0.03)	(0.03)	(0.03)	(0.03)
Minimal denial	1.02	1.02	1.02	1.02	1.02	1.02	1.03	1.03	1.03	1.04	1.04	1.03	1.02	1.02	1.02
	(0.03)	(0.03)	(0.03)	(0.03)	(0.03)	(0.03)	(0.03)	(0.03)	(0.03)	(0.03)	(0.03)	(0.03)	(0.03)	(0.03)	(0.03)
Constant	0.18**	0.19**	0.18**	0.19**	0.19**	0.19**	0.18**	0.17**	0.16**	0.18**	0.18**	0.18**	0.18**	0.18**	0.18**
	(0.12)	(0.12)	(0.11)	(0.12)	(0.12)	(0.12)	(0.11)	(0.11)	(0.10)	(0.11)	(0.11)	(0.11)	(0.12)	(0.11)	(0.11)
Observations	1,254	1,254	1,254	1,254	1,254	1,254	1,254	1,254	1,254	1,254	1,254	1,254	1,254	1,254	1,254

*p<0.05, **p<0.01, ***p<0.001

CM = childhood maltreatment

[a] CM sub-indexes have been standardized

[b] Each set of columns comprises models that include only the CM sub-index given in the column heading.

[c] For overall physiological dysregulation, cardiometabolic risk, and HPA/SNS function, the coefficients (not shown) imply that dysregulation increases with age up to a point and then declines at older ages (i.e., the coefficient for the linear age term is positive (IRR>1.0) but the coefficient for the quadratic age term is negative (IRR<1.0)). In the case of inflammation, the coefficient for the linear age term is negative (IRR<1.0) but the coefficient for the quadratic age term is positive (IRR>1.0), implying that inflammation decreases with age to a point but increases after that.

Notes: SES=Socioeconomic status; Models: (1) main effects; (2) main effects + sex interactions; (3) main effects + race interactions
