# Supplementary material for: Exposure to childhood maltreatment predicts adult physiological dysregulation, particularly inflammation
Source: PLoS One. 2023 Nov 30;18(11):e0294667. doi: 10.1371/journal.pone.0294667 (PMC10688890; doi:10.1371/journal.pone.0294667)
Supplement: Supplementary file 10 [file pone.0294667.s010.docx]

S10 Table: Incidence-rate ratios (standard errors) from negative binomial (NB) or Poisson (P) regression models predicting biomarker health outcomes: Childhood maltreatment (CM) sub-indexes (N=1254)

	Overall Physiological Dysregulation (NB)			Cardiometabolic Risk (P)			Inflammation (NB)			HPA/SNS Function (P)		
VARIABLES	(1)	(2)	(3)	(1)	(2)	(3)	(1)	(2)	(3)	(1)	(2)	(3)
CM sub-indexes [a]												
Emotional Abuse	1.02	1.07	1.00	1.04	1.07	1.03	1.04	1.10	1.02	0.96	0.97	0.92
	(0.02)	(0.04)	(0.03)	(0.03)	(0.05)	(0.04)	(0.05)	(0.09)	(0.05)	(0.05)	(0.09)	(0.05)
Physical Abuse	1.01	1.03	1.03	1.01	1.04	1.04	1.06	1.01	1.06	0.97	1.00	0.96
	(0.02)	(0.03)	(0.02)	(0.03)	(0.04)	(0.03)	(0.04)	(0.06)	(0.05)	(0.04)	(0.08)	(0.05)
Sexual Abuse	1.02	0.92*	0.99	1.01	0.94	1.01	1.00	0.87	0.93	1.04	0.93	1.03
	(0.02)	(0.04)	(0.02)	(0.02)	(0.04)	(0.03)	(0.03)	(0.07)	(0.04)	(0.03)	(0.09)	(0.04)
Emotional Neglect	1.03	1.03	1.03	1.00	1.01	1.00	1.04	1.04	1.06	1.07	1.10	1.10
	(0.02)	(0.03)	(0.03)	(0.03)	(0.04)	(0.03)	(0.04)	(0.07)	(0.05)	(0.05)	(0.08)	(0.06)
Physical Neglect	0.98	0.98	1.00	0.98	0.95	0.99	1.00	1.10	1.06	0.97	0.95	0.99
	(0.02)	(0.03)	(0.02)	(0.02)	(0.03)	(0.03)	(0.04)	(0.06)	(0.05)	(0.04)	(0.07)	(0.05)
Female	0.89***	0.91**	0.90***	0.66***	0.67***	0.66***	1.11	1.14*	1.12	1.53***	1.56***	1.54***
	(0.03)	(0.03)	(0.03)	(0.02)	(0.03)	(0.02)	(0.06)	(0.07)	(0.06)	(0.09)	(0.10)	(0.09)
Race (White omitted)												
Black	1.21***	1.21***	1.23***	1.16**	1.17**	1.20***	1.49***	1.47***	1.50***	0.98	0.98	0.99
	(0.05)	(0.05)	(0.05)	(0.06)	(0.06)	(0.06)	(0.10)	(0.10)	(0.10)	(0.08)	(0.08)	(0.08)
Other	1.14	1.14	1.18*	1.06	1.06	1.11	1.34*	1.35*	1.36*	1.12	1.12	1.15
	(0.09)	(0.09)	(0.10)	(0.10)	(0.10)	(0.11)	(0.19)	(0.19)	(0.22)	(0.18)	(0.18)	(0.20)
Sex and CM sub-index interactions												
Female X Emotional Abuse		0.94			0.96			0.93			0.99	
		(0.05)			(0.06)			(0.09)			(0.11)	
Female X Physical Abuse		0.97			0.93			1.08			0.95	
		(0.04)			(0.05)			(0.08)			(0.09)	
Female X Sexual Abuse		1.13**			1.11*			1.17			1.14	
		(0.05)			(0.06)			(0.10)			(0.12)	
Female X Emotional Neglect		0.99			0.98			1.01			0.97	
		(0.04)			(0.05)			(0.08)			(0.09)	
Female X Physical Neglect		1.01			1.07			0.87*			1.03	
		(0.04)			(0.05)			(0.06)			(0.09)	
Race and CM sub-index interactions												
Black X Emotional Abuse			1.07			1.08			1.00			1.20
			(0.07)			(0.08)			(0.11)			(0.15)
Other X Emotional Abuse			1.08			1.08			1.10			1.10
			(0.12)			(0.16)			(0.21)			(0.24)
Black X Physical Abuse			0.89*			0.83**			0.94			0.97
			(0.04)			(0.05)			(0.08)			(0.10)
Other X Physical Abuse			0.98			1.00			0.91			1.03
			(0.11)			(0.15)			(0.21)			(0.25)
Black X Sexual Abuse			1.10**			1.06			1.24***			1.05
			(0.04)			(0.05)			(0.08)			(0.08)
Other X Sexual Abuse			0.84			0.91			0.79			0.75
			(0.09)			(0.12)			(0.17)			(0.16)
Black X Emotional Neglect			1.00			1.02			1.00			0.96
			(0.05)			(0.07)			(0.09)			(0.10)
Other X Emotional Neglect			0.90			0.89			0.87			0.91
			(0.11)			(0.15)			(0.20)			(0.22)
Black X Physical Neglect			0.94			0.99			0.87			0.92
			(0.04)			(0.06)			(0.07)			(0.09)
Other X Physical Neglect			0.98			0.90			1.05			1.05
			(0.09)			(0.11)			(0.17)			(0.17)
Controls												
Age[b]	1.01	1.01	1.01	1.03*	1.03*	1.04**	0.97	0.97	0.98	1.03	1.03	1.03
	(0.01)	(0.01)	(0.01)	(0.01)	(0.01)	(0.01)	(0.02)	(0.02)	(0.02)	(0.02)	(0.02)	(0.02)
Age squared[b]	1.00	1.00	1.00	1.00*	1.00*	1.00*	1.00*	1.00*	1.00	1.00	1.00	1.00
	(0.00)	(0.00)	(0.00)	(0.00)	(0.00)	(0.00)	(0.00)	(0.00)	(0.00)	(0.00)	(0.00)	(0.00)
Childhood SES	0.95**	0.95**	0.95**	0.94**	0.94**	0.94**	0.97	0.97	0.97	0.96	0.96	0.96
	(0.02)	(0.02)	(0.02)	(0.02)	(0.02)	(0.02)	(0.03)	(0.03)	(0.03)	(0.03)	(0.03)	(0.03)
Minimal denial score	1.02	1.02	1.02	1.00	1.00	1.00	1.05	1.06	1.06	1.04	1.04	1.04
	(0.02)	(0.02)	(0.02)	(0.02)	(0.02)	(0.02)	(0.03)	(0.03)	(0.03)	(0.03)	(0.04)	(0.04)
Constant	2.84***	2.84***	2.53**	1.15	1.14	1.06	1.93	1.94	1.56	0.19**	0.19**	0.17**
	(0.88)	(0.88)	(0.79)	(0.46)	(0.45)	(0.42)	(1.10)	(1.10)	(0.89)	(0.12)	(0.12)	(0.11)

*** p<0.001, ** p<0.01, * p<0.05

[a] The CM sub-indexes were standardized.

[b] IRR for the CM sub-indexes from models unadjusted for controls weare as follows:
Overall physiological dysregulation: EA=0.98 (0.02); PA=1.03 (0.02); SA=1.01 (0.02); EN=1.02 (0.02); PN=1.02 (0.02)
Cardiometaboblic risk: EA=0.98 (0.03); PA=1.06* (0.03); SA=0.98 (0.02); EN=1.02(0.03); PN=1.00 (0.02)
Inflammation: EA=1.01 (0.05); PA=1.06 (0.04); SA=1.02 (0.03); EN=1.00 (0.04); PN=1.05 (0.04)
HPA/SNS: EA=0.96 (0.05); PA=0.90* (0.04); SA=1.09** (0.03); EN=1.03 (0.04); PN=1.02 (0.04)

[c] For overall physiological dysregulation, cardiometabolic risk, and HPA/SNS function, the coefficients (not shown) imply that dysregulation increases with age up to a point and then declines at older ages (i.e., the coefficient for the linear age term is positive (IRR>1.0) but the coefficient for the quadratic age term is negative (IRR<1.0)). In the case of inflammation, the coefficient for the linear age term is negative (IRR<1.0) but the coefficient for the quadratic age term is positive (IRR>1.0), implying that inflammation decreases with age to a point but increases after that.

Notes: HPA/SNS=Hypothalamic-Pituitary Axis and Sympathetic Nervous system; SES=Socioeconomic status
Models: (1) main effects; (2) main effects + sex interactions; (3) main effects + race interactions
